# Inhibitory Control in Excessive Social Networking Users: Evidence From an Event-Related Potential-Based Go-Nogo Task

**DOI:** 10.3389/fpsyg.2019.01810

**Published:** 2019-08-07

**Authors:** Qiufeng Gao, Ge Jia, Jun Zhao, Dandan Zhang

**Affiliations:** ^1^Department of Sociology, Shenzhen University, Shenzhen, China; ^2^College of Psychology, Shenzhen University, Shenzhen, China; ^3^Shenzhen Key Laboratory of Affective and Social Cognitive Science, Shenzhen University, Shenzhen, China

**Keywords:** excessive use of social networking site, inhibitory control, event-related potential, Go-Nogo, N2, P3

## Abstract

Inhibitory control is a core executive function module that monitors and suppresses inappropriate behavior. Inhibitory deficits have been observed in different addiction types (e.g., smoking, alcohol, drug and gambling). The excessive use of social networking sites (SNSs) has attracted increasing attention; however, it is unknown whether inhibitory control is impaired in excessive SNS users. This study used event-related potentials in an SNS-related Go-Nogo task to investigate inhibitory control in excessive SNS users. Although the behavioral data did not show any significant differences between groups, the N1 amplitude was larger following SNS images than control images in excessive SNS users. Furthermore, excessive users showed larger N2 amplitude and smaller Nogo-P3 amplitude than non-excessive users irrespective of stimuli. These findings suggested that excessive SNS users are inefficient in allocating monitoring resources in the Go-Nogo task (reflected by enhance N2) and show difficulty in late inhibitory control procedure (reflected by reduced Nogo-P3) compared to non-excessive users. Also, excessive SNS users pay more attention to SNS-related images compared to non-SNS-related images (reflected by the N1). Interventions for this specific population should focus on limiting exposure to SNS cues and enhancing inhibitory control.

## Introduction

The dramatic increase in smartphone use in recent years has led to significant societal changes, and such technology has become indispensable (Oulasvirta et al., 2012). There is no doubt that smartphones provide numerous advantages. However, the disadvantages of smartphones, such as smartphone addiction and overuse, have also been examined. According to previous studies, game overuse is the most common subtype of Internet overuse. Similarly, smartphone overuse has mainly focused on social network addiction (Petry and O’Brien, 2013; Jeong et al., 2016). Social networking applications, such as Facebook, Twitter and YouTube, have entirely changed our traditional way of communication (Yu et al., 2011) and allow us to communicate with each other anytime and anywhere with no regard for barriers of time and space (Billieux et al., 2015).

In China, WeChat and QQ have been the most popular social platforms, especially for youth (Hou et al., 2017). According to the 2017 earnings report of Tencent (the company that created WeChat and QQ), the monthly number of living users of WeChat has reached approximately 1,000 million, and for QQ, this number was approximately 800 million^1^. The latest China Internet network development statistics report (released by China Internet Network Information Center at http://www.cnnic.net.cn/) showed that 18- to 24-year-olds have become the main user group for social networking. Taken together, although social networks facilitate communication between people, excessive use of social networking sites (SNSs) also creates a number of problems (Zheng and Lee, 2016), such as distracted attention in learning environments, poor academic performance, bad time management (Kirschner and Karpinski, 2010; Hong et al., 2014), poor psychological consequences (i.e., low self-evaluation and negative emotions; Kormas et al., 2011; Deters and Mehl, 2013; Lemola et al., 2015; Sampasa-kanyinga and Hamilton, 2015), and poor physical outcomes (Douglas et al., 2008; Milani et al., 2009; Ross et al., 2009; Gosling et al., 2011). Moreover, Oulasvirta et al. (2012) suggested that excessive use of SNSs is an abnormal habit and SNS-related content has become an extremely strong cue for compulsively checking the device. These abnormal habits repeatedly triggered by cues have been found to reduce the intrinsic control of an individual. Hence, we hypothesized that there might be a difference in inhibitory control between excessive SNS users and non-excessive users. However, there have been no empirical studies on inhibitory control in excessive users of SNS to date. Therefore, the purpose of this paper was to examine the role of inhibitory control in this population.

In previous studies on addiction, inhibitory control has attracted widespread attention. Inhibitory control is the ability to restrain from engaging in behaviors that are inappropriate or not currently required. This cognitive ability is essential for individuals to make flexible and goal-directed decisions based on environmental changes (Kerns et al., 2004). Inhibitory control enables us to choose how we react, rather than becoming habitual, impulsive, thoughtless creatures (Diamond, 2013). This ability is closely related to various aspects of life, such as physical and mental health, quality of life, academic performance, work achievement and interpersonal relationships (Pessoa et al., 2012; Cotrena et al., 2015; Lim et al., 2016). Previous research has found that inhibitory control deficits are inseparable from alcohol abuse, drug addiction, attention deficits, etc. (Yongliang et al., 2000; Kamarajan et al., 2005; Luijten et al., 2011). Since excessive users of mobile phones may develop some characteristics similar to substance dependence, e.g., tolerance, withdrawal, mood modification, conflict and relapse (van Rooij et al., 2010; Weinstein and Lejoyeux, 2010; Kwon et al., 2013; Lin et al., 2014; Matar Boumosleh and Jaalouk, 2017), we hypothesized that there might be impaired inhibitory control in excessive SNS users, who represent a common subtype of smartphone overusers.

The Go-Nogo task is a frequently used paradigm in the investigation of inhibitory control (Spinella, 2002; Dinn et al., 2004; Gotlib et al., 2004; Monterosso et al., 2005; Reynolds et al., 2007; Luijten et al., 2011; Messerotti Benvenuti et al., 2015, 2017; Mennella et al., 2017). The paradigm requires participants to respond as quickly as possible when the “Go” stimuli are presented, while participants need to inhibit their response with the presentation of “Nogo” stimuli (Jonkman, 2006; Kirmizi-Alsan et al., 2006). In other words, participants will show inhibitory control in the “Nogo” condition. According to fMRI studies, the anterior cingulate cortex (ACC), orbitofrontal cortex and pre-supplementary area (preSMA) are core regions associated with inhibition control during the Go/NoGo task (Braver et al., 2001; Luijten et al., 2014; Palermo et al., 2018a,b). Event-related potentials (ERPs) with a high temporal resolution have been recommended as a sensitive method to investigate response activation and response inhibition. Previous studies have mainly focused on the ERP components of Nogo-N2 related to conflict detection and Nogo-P3 in relation to response inhibition (Dong et al., 2010; Huster et al., 2013; Detandt et al., 2017). The Nogo-N2 displays an enhanced negative amplitude at 200–400 ms after the presentation of the Nogo stimulus and is maximal in the prefrontal lobe (Eimer, 1993; Huster et al., 2013). Source localization or fMRI-ERP combined data analyses have demonstrated that the Nogo-N2 is likely to be associated with neural activity in the orbitofrontal area and the ACC (Van Veen and Carter, 2002; Ullsperger and von Cramon, 2004; Luus et al., 2007; Luijten et al., 2014). The Nogo-P3 appears 300–600 ms after the presentation of the stimulus (Bokura et al., 2001; Huster et al., 2013; Luijten et al., 2014; D’Hondt and Maurage, 2017). The neural source of this ERP component is considered to be located in the preSMA region (Albert et al., 2013).

Previous studies focusing on inhibitory control used addiction-unrelated stimuli in the Go-Nogo paradigm to explore inhibitory control in individuals with addiction-related disorders or impairments. In particular, Kamarajan et al. (2005) investigated the response inhibition in alcoholics and found a decreased P3 in alcoholics during the Go-Nogo task compared to healthy controls. Similarly, Evans et al. (2009) observed that smokers had smaller Nogo-P3 amplitude relative to nonsmokers. Furthermore, Yin et al. (2016) investigated adolescent smokers and found reduced NoGo-P3 amplitude in these subjects relative to nonsmokers. However, Dong et al. (2010) found that excessive Internet users had larger Nogo-P3 amplitudes and smaller Nogo-N2 amplitudes (see also Zhou et al., 2010) compared to control subjects, whereas Littel et al. (2012) found no differences in Nogo-N2 and Nogo-P3 amplitudes between excessive users and controls. It has been suggested, however, that there might exist a relationship between addiction-related cues and processes of executive functioning (Jentsch and Taylor, 1999; Dawe et al., 2004). In recent years, many studies have revealed that a critical factor in the maintenance and relapse of addictive behaviors is cue-induced craving (Sinha and Li, 2007; Ashrafioun and Rosenberg, 2012). In other words, addiction-related cues are more likely to attract the attention of individuals with addiction and further generate poor inhibitory control performance (Franken, 2003; Olmstead, 2006; Field and Cox, 2008). Consistent with this concept, a modified Go-Nogo paradigm has been adopted to investigate inhibitory control using addiction-related stimuli.

Luijten et al. (2016) used smoking-related cues in Go-Nogo task and examined the association between smoking relapse and ERP bio-markers, which found that smaller inhibitory control P3 amplitudes could predict an increased relapse risk, thus suggesting that smokers with a large relapse risk have seriously impaired inhibitory control functions. Another study accomplished by Kreusch et al. (2014) investigated whether inhibitory control in alcohol dependent individuals was especially impaired when assessed with alcohol-related cues in a Go-Nogo task, and they found that heavy drinkers showed larger Nogo-N2 amplitude than light drinkers in the alcohol modified Go-Nogo task. Detandt et al. (2017) examined whether the inhibitory control function was more seriously impaired when presenting smoking-related background compared to nonsmoking-related background for smokers, and they found that Nogo-N2 latencies were shorter in smokers than nonsmokers independent of stimuli type, suggesting that smokers had an overall impairment in inhibition. However, it is worth noting that the subjects who smoked exhibited a larger Nogo-P3 amplitude in response to the smoking-related stimuli relative to other stimuli, indicating that smokers allocated more inhibitory sources to the background cues related to smoking. Chen et al. (2016) investigated whether inhibitory control in smartphone excessive users was impaired when presenting smartphone-related cues in a Go-Nogo task, and they found that excessive users displayed a larger Nogo-N2 amplitude than controls, although differences in Nogo-P3 were not observed between the groups. In their study, the stimuli were images associated with SNSs while the criteria for excessive users were based on the Smartphone Addiction Inventory Scale; thus, the stimuli and participants were somewhat mismatched. Therefore, the current study used the SNS Excessive Use Scale to provide more reasonable selection criteria for excessive SNSs users. In addition, participants were instructed to respond to stimulus features unrelated to addiction (i.e., respond to the color of image frame) in Chen et al. (2016). In this study, we asked participants to directly focus on SNS-related cues to explicitly examine their inhibitory control.

Although there have been many studies investigating the relationship between inhibitory control and various forms of addictive behaviors, such as alcohol abuse, drug addiction, smoking addiction, Internet addiction, and smartphone addiction (Franken, 2003; Olmstead, 2006; Field and Cox, 2008; Luijten et al., 2011; Detandt et al., 2017), studies on the excessive use of SNSs that are based on mobile social networking applications are rare. This study was performed to address this gap. The present study adopted a SNS-related Go-Nogo paradigm combined with the ERP technique to examine the impaired inhibitory control in excessive SNS users and to reveal whether there is any difference in brain response between excessive SNS users and control subjects in the context of SNS cues. To this end, two types of stimuli (SNS-related images and control images) were used. Consistent with previous related studies (Zhou et al., 2010; Littel et al., 2012), we expected that excessive SNS users would respond faster to SNS-related than SNS-unrelated Go trials and show lower accuracy to SNS-related than SNS-unrelated Nogo trials as compared to the controls. For the ERP indexes, we hypothesized that compared to the controls, excessive SNS users would have an inhibitory dysfunction with larger Nogo-N2 amplitudes (Kreusch et al., 2014; Chen et al., 2016; but see Dong et al., 2010; Zhou et al., 2010). Since most related studies indicated that small P3 amplitudes in addicts are a marker for impaired inhibitory control (Kamarajan et al., 2005; Evans et al., 2009; Luijten et al., 2016; Yin et al., 2016; but see Dong et al., 2010; Detandt et al., 2017), we hypothesized that excessive SNS users would have an inhibitory deficit with smaller Nogo-P3 amplitude compared to the controls. Furthermore, we suggested that SNS-related images will be more sensitive indicators for the detection of impaired inhibitory control in addicts; therefore, it is expected that excessive SNS users would show larger Nogo-N2 amplitude and smaller Nogo-P3 amplitude in response to SNS-related images vs. SNS-unrelated images as compared to control group. In addition, early attentional enhancement has been observed in alcohol abuse at the presentation of alcohol-related stimuli (Petit et al., 2012; Matheus-Roth et al., 2016). Accordingly, we also examined the frontal N1 component to determine whether excessive users would show an early attentional enhancement with exposure to SNS images.

## Materials and Methods

### Participants

Two thousand students were recruited on-line at Shenzhen University to complete a questionnaire about SNS usage. The questionnaire consisted of the SNS Excessive Use Scale and three pairs of lie detection questions (in order to ensure the credibility of the questionnaire). The exclusion criteria for valid questionnaires were as follows: (1) response time less than 10 min or more than 60 min; (2) inconsistent answers for more than two lie detection questions; and (3) individuals with a history of mental illness or alcohol/drug abuse. Finally, 1,431 valid questionnaires were obtained.

The SNS Excessive Use Scale was modified from the WeChat Excessive Use Scale (Hou et al., 2017), which consisted of 10 items and included three factors (mood modification, salience and conflict). Each item was scaled to a five-point Likert scale (1 for not at all and 5 for always). The original internal consistency of the scale was 0.896 (Cronbach’s alpha). In this study, individuals with total scores lower than 6.9 (mean − 1.96 standard deviation) were considered as non-excessive users, whereas individuals with total scores higher than 39.9 (mean + 1.96 standard deviation) were considered as excessive SNS users (Hou et al., 2017). Based on the threshold of the SNS Excessive Use Scale, 50 participants were recruited for the ERP experiment, with 25 excessive SNS users and 25 non-excessive users. Among them, seven participants were excluded due to invalid segments of ERP data. Finally, 43 participants, including 23 excessive SNS users (11 females; average age = 19 ± 1.0 years; SNS Excessive Use Scale score = 42.3 ± 2.8) and 20 non-excessive users (10 females; average age = 20 ± 1.0 years; SNS Excessive Use Scale score = 4.4 ± 2.0), were included in the analyses. All participants were right-handed and had normal or corrected visual acuity. In addition, their age and education level were matched.

Participants signed informed consent forms at the start of the experiment and received appropriate payment after the experiment. The experimental protocol was approved by the Ethics Committee of Shenzhen University.

### Stimuli

**Figure 1:**
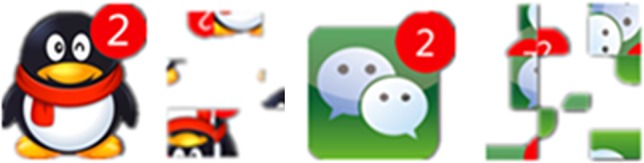
SNS images and control images used in the present study. If the social network image represented the Go condition, then the control image represented the Nogo condition, and vice versa.

The stimuli consisted of SNS-related images (i.e., WeChat and QQ logos) and control images. To reduce the physical differences between social network cues and irrelevant cues, we divided the SNS-related images into nine sections and then randomly combined them to create the control images (Figure 1). We used two stimuli (i.e., WeChat and QQ logos) in order to prevent a ceiling effect.

During the task, a fixation point was first presented in the center of the computer monitor with a visual angle of 0.5° × 0.5°, and one image was displayed with a visual angle of 3.0° × 3.0°. The image was presented randomly with equal probability within the Go (*p* = 0.8, i.e., *p* = 0.4 for each image) and Nogo conditions (*p* = 0.2, i.e., *p* = 0.1 for each image). Stimuli were presented on a white background.

### Procedure

Participants were seated in a comfortable experimental laboratory and exposed to limited sound and appropriate light. The stimuli were presented on a computer screen approximately 100 cm away from the participant. The experimental program was designed and the behavior data were collected using E-prime 2.0.

First, the fixation point in the center of the computer monitor was presented for 200 ms and then one image was presented for 1,500 ms. Participants were asked to make a decision based on the presented image according to the instructions. For the “Go” stimulus, participants needed to press the “J” key as quickly as possible to make it disappear. For the “Nogo” stimulus, participants should not respond until it disappear after 1,500 ms. This study made the “Go” stimulus disappear upon key pressing since this setting could indicate a successful button press and potentiate the behavioral expression of readiness (see also Buodo et al., 2017). After presentation of the stimulus, a blank screen appeared and lasted for 1,500 ms. Then, the next trial was initiated. The entire experiment consisted of 2 blocks, with 200 trials in each block. The Go and Nogo trials were presented randomly at a ratio of 4:1 in each block. It is worth mentioning that the task requirements between the two blocks were different. In one block, the Go targets were SNS-related images while in another block the Go targets were control images. The order of the two blocks were counterbalanced across participants. The entire experiment lasted approximately 20 min.

### Event-Related Potential Recording and Analysis

The EEG data were recorded using a 64-channel amplifier (Brain Products, Gilching, Germany) with a sample rate of 500 Hz. The vertical electrooculogram (EOG) was collected at the external canthi of both eyes. All signals were referenced to the left mastoids. The scalp impedances were less than 5 kΩ.

This study used Brain Vision Analyzer (v.2.1, Brain Products, Gilching, Germany) to analyze the data. The recorded EEG data were first referenced to linked mastoids. Then, an independent component analysis was performed for the correction of eye movements and eye blinks. Subsequently, the data were filtered with a band-pass of 0.01–30 Hz. The filtered data were segmented in 1200 ms epochs in which the initial 200 ms of prestimulus interval served as baseline. Data epochs exceeding ±80 μV were removed. In the end, the signals related to target stimulation were averaged. In our study, incorrect responses, i.e., no response in Go trials or false alarms in Nogo trials, were excluded from the analysis.

The present study focused on the mean amplitudes and peak latencies of the N2 and P3 components associated with inhibitory control. The N2 was measured using the mean amplitude and peak latency at the electrode sites of the Fz and FCz within a time window of 180–300 ms. P3 was measured using the mean amplitude and peak latency at the Cz, CPz and Pz electrode sites within a time window of 350–500 ms. Peak latencies were manually detected in individual ERP waveforms within the analysis window. In addition, only one-quarter of the Go trials were randomly selected for the ERP analysis to balance the number of Go and Nogo trials. N1 was also included in the analysis, and its amplitude was averaged within a time window of 80–130 ms across the Fz and FCz sites. Baseline-to-peak amplitudes were calculated for the N1 and P3, and peak-to-peak amplitudes (i.e., the amplitude difference between the associated peak and the previous peak) were calculated for the frontal N2 to isolate the amplitude contribution of this component from a prior component (i.e., frontal P1; see also Zhang et al., 2013) since amplitude differences between conditions were observed in the P1 component.

### Statistics

All data analyses were performed using SPSS 22.0, and the significance level was set at 0.05. All measures were first tested for normal distribution using the Kolmogorov–Smirnov method. A Box-Cox transformation was performed to normalize the data if necessary. ERP amplitudes (N1, N2, and P3) were analyzed using a three-way repeated-measures ANOVA, with trial type (Go, Nogo) and image type (SNS image, control image) as the within-subject factors and group (excessive users, non-excessive users) as the between-subject factor. Reaction times (RTs) were analyzed using a two-way repeated-measures ANOVA with image type and group as factors. The accuracy rate was first normalized using the Box-Cox transformation and then analyzed using a two-way repeated-measures ANOVA, with image type and group as factors. In addition, based on signal detection theory, four indicators (the hit rate, false alarm rate, miss rate and correct rejection) were analyzed using a two-way repeated-measures ANOVA, with image type and group as factors. *Post hoc* tests were conducted when the main effects were significant. When the interactions were significant, simple effects analysis was performed.

## Results

### Behavioral Results

#### Accuracy Rate

The main effect of trial type was significant [*F*(1, 41) = 175.4, *p* < 0.001, $\eta_{p}^{2}$ = 0.811]: the ACC was lower in the Nogo trials than the Go trials. The main effect of group was not significant [*F*(1, 41) = 0.3, *p =* 0.586, $\eta_{p}^{2}$ = 0.007]. The group × image type [*F*(1, 41) = 1.9, *p =* 0.179, $\eta_{p}^{2}$ = 0.040], group × trial type [*F*(1, 41) = 0.2, *p =* 0.640, $\eta_{p}^{2}$ = 0.005], trial type × image type [*F*(1, 41) = 1.2, *p =* 0.293, $\eta_{p}^{2}$ = 0.021], and the group × image × trial type interactions [*F*(1, 41) = 0.3, *p =* 0.573, $\eta_{p}^{2}$ = 0.004] were not significant.

Considering that the proportion of trials for Go and Nogo was 4:1, the difference in number of trials may have led to differences in accuracy; therefore, we performed two independent RM-ANOVA tests for the trial type (Go, Nogo). However, neither the main effect of group nor the interactions between group × image type, group × trial type were not significant.

Based on signal detection theory, the hit rate, false alarm rate, miss rate and correct rejection rate were measured. However, neither the main effects of trial type and group nor the interaction effect was significant for the four indicators.

#### Reaction Times

The two-way repeated-measures ANOVA showed that the main effect of image type was significant [*F*(1, 41) = 27.5, *p* < 0.001, $\eta_{p}^{2}$ = 0.407], with quicker responses observed following the SNS images than the control images. However, the main effect of group was not significant [*F*(1, 41) = 0.8, *p =* 0.375, $\eta_{p}^{2}$ = 0.02] and the group × image type was not significant [*F*(1, 41) = 2.6, *p =* 0.115, $\eta_{p}^{2}$ = 0.061]. The descriptive statistics of the behavioral indexes are listed in Table 1.

**Table 1:** Descriptive statistics of the behavioral data (mean ± standard deviation).

Group	Accuracy rate				RT	
	SNS Go	Control Go	SNS Nogo	Control Nogo	SNS Go	Control Go
Excessive	0.99 ± 0.01	0.99 ± 0.01	0.84 ± 0.08	0.86 ± 0.08	139 ± 32	151 ± 37
Non-excessive	0.99 ± 0.01	0.99 ± 0.01	0.86 ± 0.08	0.86 ± 0.08	144 ± 26	162 ± 34

### Event-Related Potential Results

No significant difference was found in the peak latencies of the N1, N2 and P3 components across conditions. The statistical results of the mean amplitudes are reported.

#### N1 Component

**Figure 2:**
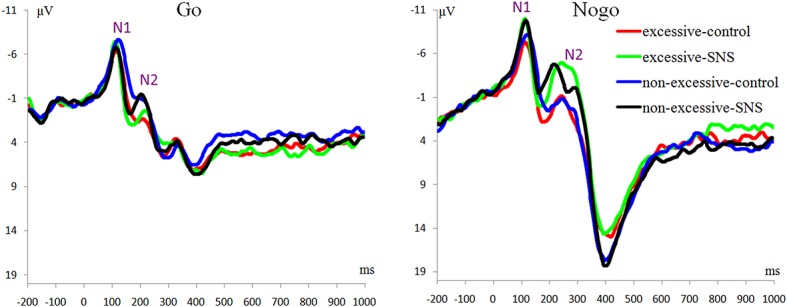
ERP waveforms averaged across Fz and FCz.

The main effect of trial type was significant [*F*(1, 41) = 46.6, *p* < 0.001, $\eta_{p}^{2}$ = 0.532] and the amplitude of N1 was larger on the Nogo trials than on the Go trials (Nogo = −7.51 ± 3.04 μV, Go = −5.65 ± 2.95 μV). The main effect of image type was significant [*F*(1, 41) = 12.3, *p* = 0.001, $\eta_{p}^{2}$ = 0.231] and the amplitude of N1 was larger for the SNS image than for the control image (SNS image = −7.02 ± 3.13 μV, control image = −6.24 ± 2.84 μV). More importantly, the interaction of image type by group was significant [*F*(1, 41) = 7.7, *p* = 0.008, $\eta_{p}^{2}$ = 0.159; Figure 2]. Further simple effect analyses indicated that a larger N1 amplitude was induced with the SNS image than the control image in the excessive SNS users [*F*(1, 41) = 21.3, *p* < 0.001, $\eta_{p}^{2}$ = 0.342; SNS image = −7.10 ± 3.59 μV, control image = −5.73 ± 3.07 μV], whereas significant differences were not observed for the non-excessive users [*F*(1, 41) = 0.8, *p* = 0.412, $\eta_{p}^{2}$ = 0.009].

#### N2 Component

The main effect of trial type was significant [*F*(1, 41) = 66.6, *p <* 0.001, $\eta_{p}^{2}$ = 0.619]: the amplitude of N2 was larger on the Nogo trials than on the Go trials (Nogo = −3.31 ± 3.77 μV, Go = −0.18 ± 2.94 μV). The main effect of group was significant [*F*(1, 41) = 4.2, *p* = 0.047, $\eta_{p}^{2}$ = 0.093] and the amplitude of N2 was larger in the excessive users than in non-excessive users (excessive users = −2.54 ± 2.71 μV, non-excessive users = −0.66 ± 3.30 μV; Figure 2). The main effect of image type was significant [*F*(1, 41) = 18.3, *p* < 0.001, $\eta_{p}^{2}$ = 0.309] and the amplitude of N2 was larger for the SNS image than for the control image (SNS image = −2.55 ± 3.23 μV, control image = −0.78 ± 3.54 μV). However, the group × image type, group × trial type, the image type × trial type and group × trial type × image type interactions were not significant.

#### P3 Component

**Figure 3:**
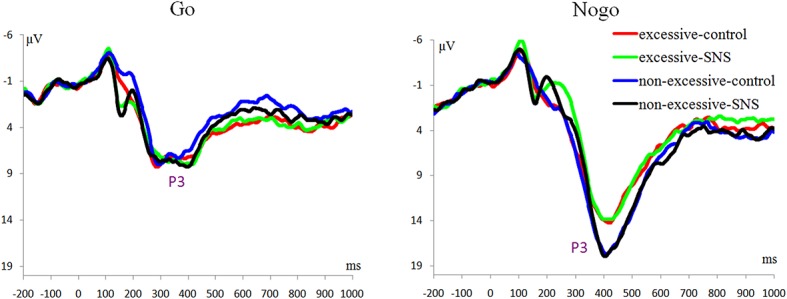
ERP waveforms averaged across Pz and CPz.

**Figure 4:**
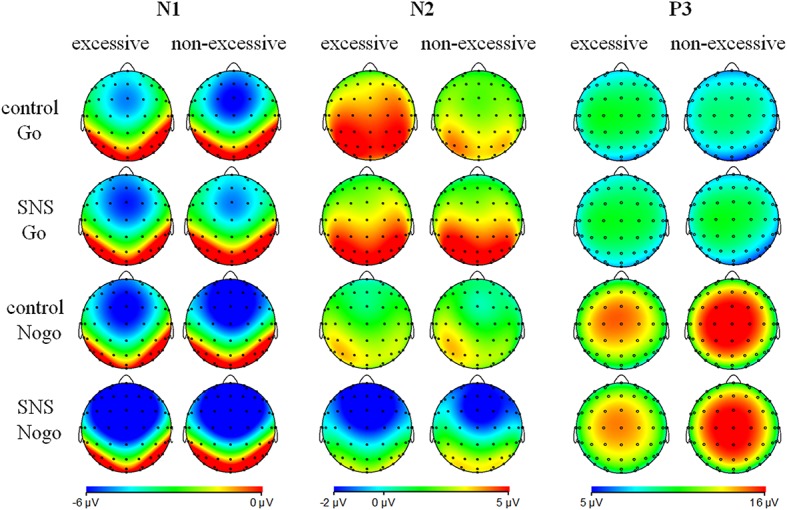
Topographic maps of the N1 (80–130 ms), N2 (180–300 ms) and P3 components (350–500 ms).

The main effect of trial type was significant [*F*(1, 41) = 163.5, *p* < 0.001, $\eta_{p}^{2}$ = 0.800] and the amplitude of P3 was larger on the Nogo trials than on the Go trials (Nogo = 14.08 ± 4.99 μV, Go = 6.26 ± 2.88 μV). The group × trial type interaction effect was significant [*F*(1, 41) = 9.3, *p* = 0.004, $\eta_{p}^{2}$ = 0.185; Figure 3]. A further simple effect analysis indicated that the P3 amplitude in the Nogo condition was lower in the excessive users than the non-excessive users [*F*(1, 41) = 4.7, *p* = 0.035, $\eta_{p}^{2}$ = 0.103; excessive users = 12.60 ± 4.10 μV, non-excessive users = 15.78 ± 5.46 μV] while the group effect was not significant in the Go condition [*F*(1, 41) = 0.3, *p =* 0.581, $\eta_{p}^{2}$ = 0.005]. However, the main effect of group or image type was not significant. The group × image type, image type × trial type and group × trial type × image type interactions were not significant. Topographic maps of the N1, N2 and P3 components are shown in Figure 4.

## Discussion

The present study investigated impaired inhibitory control in excessive SNS users and attempted to verify whether there were any differences in brain activity between excessive SNS users and non-excessive users in response to SNS-related cues in a Go-Nogo task combined with ERP analysis. No differences between excessive users and non-excessive users were observed at the behavioral level. Although this result is contradictory to our hypothesis, it is consistent with the findings of Dong et al. (2010). One possible reason is that the behavioral measures were not sensitive enough. Another reason may be that the method (i.e., a questionnaire) used in this study was not adequate to categorize individuals as excessive and non-excessive SNS users. However, differences were found at the electrophysiological level.

Previous studies focused on the Go-Nogo task have suggested that Nogo-N2 might reflect conflict monitoring in the early stages of inhibitory control and that the increased Nogo-N2 could be explained as a high demand for the neural resources associated with inhibitory control (Donkers and van Boxtel, 2004; Kenemans et al., 2005; Randall and Smith, 2011). In the present study, enhanced Nogo-N2 amplitudes were observed compared to the Go-N2 amplitudes, which is consistent with the notion of conflict monitoring and inhibitory control processes. Meanwhile, the main effect of group was significant: excessive SNS users exhibited larger N2 amplitudes than non-excessive users. However, the interaction effect between the group and trial type was not found, which is inconsistent with our hypothesis. When N2 is elicited in the context of tasks requiring response inhibition, this component is responsive to both activation (Go) and inhibition (Nogo) stimuli. N2 is interpreted as an index of response inhibition because this component is noticeably larger to inhibition stimuli than it is to activation stimuli (Hoyniak and Petersen, 2019). In a recent review of the N2 function (Gajewski et al., 2018), this component was found to be related to a more general mechanism of response selection, with a larger N2 indicating that the selection of the correct response is more demanding due to the conflict resolution; consequently, N2 is usually larger on task-switch than non-switch conditions (Gajewski et al., 2018). Consistent with this idea, the current finding of the larger N2 amplitudes in excessive SNS users might due to their higher sensitivity or familiarity with a task-switch environment, and this cognitive characteristic has also been observed in media multi-taskers (Ophir et al., 2009) and smartphone excessive users (Chen et al., 2016).

Regarding the P3 findings, previous studies have suggested that Nogo-P3 could be considered as an index for the inhibition process in the late stage and closely connected to the actual inhibition of the motor system (Dimoska et al., 2006; Kok et al., 2010; Luijten et al., 2014). In the present study, we found that the interaction between group and trial type was significant, demonstrating reduced Nogo-P3 amplitude in excessive SNS users compared to controls. It is worth noting that the reduced P3 amplitude was observed in Nogo trials independent of image type, which suggested that excessive SNS users had an overall impairment in inhibition. This result is slightly different from our hypothesis. The negative result regarding the three-way interaction might be due to the small number of SNS stimuli (only two SNS-related stimuli), which may weaken the effect of conflict with the repetition of trials (e.g., Luijten et al. (2011) used 112 smoking-related images). However, the current result is consistent with previous findings related to inhibitory control in problematic Internet users (Liu et al., 2014; Li et al., 2016), heavy smokers (Yin et al., 2016) and alcoholics (Kamarajan et al., 2005). In addition, Luijten et al. (2016) found that smaller P3 amplitudes associated with inhibitory control could predict increased smokers’ relapse risks. Nevertheless, the current Nogo-P3 finding is inconsistent with that of Dong et al. (2010) and Detandt et al. (2017), who found that excessive Internet users or smokers had larger Nogo-P3 amplitudes compared to the control subjects. In particular, Detandt et al. (2017) found in the Go-Nogo task that smokers displayed a larger Nogo-P3 amplitude than control group when Go- and Nogo-stimuli were presented with smoking-related backgrounds. However, another related study (Liu et al., 2014) found that the subjects with internet gaming disorder (compared to the control group) had lower activation at the superior parietal lobe in Nogo trials with a game-related background. Considering that Detandt et al. (2017) and Liu et al. (2014) used very similar stimulus settings but obtained opposite results, we suggest that more studies should be performed to further clarify the impaired inhibitory control in excessive alcohol, cigarette, game, Internet, and SNS users.

This study also provided electrophysiological evidence for early attentional enhancement in excessive users during relevant cue exposure. The interaction effect of the N1 component showed that excessive users are vulnerable to SNS-related cues so they were more likely to be attracted by these stimuli. Previous researchers have pointed out that the N1 or P1 could be a marker that reflects attention allocation in the early stages of cognitive processing (Wang and Bingo, 2010; Petit et al., 2012; Ernst et al., 2013; Buodo et al., 2015). Our finding is consistent with previous studies. Petit et al. (2012) found larger P1 amplitudes to alcohol-related cues compared to neutral cues in binge drinkers, indicating early enhanced perceptual processing to alcohol-related cues in drinkers. In addition, it has been reported that individuals with drug use disorders were more attracted to drug-related cues, which might further deteriorate inhibitory control performance (Olmstead, 2006). The current finding regarding the N1 component suggested that excessive users allocate more attentional resources to SNS cues and might be vulnerable to addiction relevant cues in the early visual perception stage. The cognitive processing theory (Franken, 2003) proposed that due to long-term exposure to cues related to addiction, the detection and memory related to these addiction-related cues are enhanced in addicts, which makes it difficult for addicts to allocate attention to non-addiction-related stimuli.

Certain limitations of the current study must be noted. First, considering that both excessive users and controls engage with and use SNSs in their daily lives, the distinction between excessive users and controls based on a measurement scale is not prominent compared to that in research on drug abuse. Second, impulsivity is represented by deficits in response inhibition and error processing. Therefore, it is necessary to investigate the error monitoring of the excessive users by specific tasks in combination with ERP in the future. Third, this study could not draw conclusions on causality between excessive SNS use and inhibitory control. Impaired inhibitory control may be the consequence of excessive use or reduced inhibitory control results in a tendency to become addicted to social networking. Hence, longitudinal studies, experimentally designed studies, and more complex statistical methods should be used to clarify the complicated nature of the causality in these relationships in future research. Finally, we used a relatively small sample size, which may decrease the statistical power of the study.

In conclusion, the present study indicated that excessive SNS users, exhibited an excessive or a hyper-sensitive process of response selection during the Go-Nogo task (reflected by enhanced N2) and had difficulty in motor inhibition (reflected by reduced Nogo-P3) compared to the control subjects. Also, excessive SNS users pay more attention to SNS-related compared to non-SNS-related images (reflected by the N1).

## Data Availability

All datasets generated for this study are included in the manuscript and/or the supplementary files.

## Ethics Statement

Participants signed informed consent forms at the start of the experiment and received appropriate payment after the experiment. The experimental protocol was approved by the Ethics Committee of Shenzhen University.

## Author Contributions

DZ and QG conceived the study. GJ and JZ performed the experiment. GJ and DZ analyzed the data. All authors wrote the manuscript.

### Conflict of Interest Statement

The authors declare that the research was conducted in the absence of any commercial or financial relationships that could be construed as a potential conflict of interest.

## Footnotes

^1^https://www.qq.com/pdf/2017s01.htm
